# Quantitative analysis of *Gria1*, *Gria2*, *Dlg1* and *Dlg4* expression levels in hippocampus following forced swim stress in mice

**DOI:** 10.1038/s41598-019-50689-w

**Published:** 2019-10-01

**Authors:** Florian Freudenberg

**Affiliations:** 1 Department of Psychiatry, Psychosomatic Medicine and Psychotherapy, University Hospital, Goethe University, Frankfurt, Germany; 2 Laboratory of Neural Circuits and Plasticity, University of Southern California, 3641 Watt Way, Los Angeles, CA 90089 USA

**Keywords:** Molecular neuroscience, Stress and resilience

## Abstract

AMPA receptors and interacting proteins are importantly involved in mediating stress-dependent plasticity. Previously we reported that GluA1-containing AMPA receptors and their interaction with PDZ-proteins are required for the experience-dependent expression of behavioral despair in the forced swim test. However, it is unclear if the expression of GluA1-containing AMPA receptors is affected by this type of behavior. Here we investigated in wild type mice, whether hippocampal gene or protein levels of GluA1 or associated PDZ proteins is altered following forced swim stress. We show that expression of *Dlg4* (the gene coding for PSD-95) was strongly reduced after two days of forced swimming. In contrast, levels of *Dlg1*, *Gria1*, and *Gria2* (coding for SAP97, GluA1, and GluA2 respectively) were not affected after one or two days of forced swimming. The changes in gene expression largely did not translate to the protein level. These findings indicate a limited acute effect of forced swim stress on the expression of the investigated targets and suggest that the acute involvement of GluA1-containing AMPA receptors tor forced swim behavior is a result of non-genomic mechanisms.

## Introduction

Stress is a normal reaction of an individual in response a stressor, inducing a stress response that enables the individual to adequately cope with the stressful situation^1,2^. Chronic exposure to stress or single stressful life events can result in deleterious mental conditions in humans, including depression and anxiety and the stress response in vertebrates results in activation of multiple downstream pathways (see e.g.^1–4^ for review). Importantly, stress also induces neuroplastic changes in the brain, which involves structural and molecular changes to glutamatergic synapses (reviewed in^2,5–7^). Of note, several studies have indicated changes in AMPA receptor expression, particularly of the GluA1 subunit, following different types of acute and chronic stress (reviewed in^6,8^). For example, levels of GluA1 and *Gria1* (the gene coding for GluA1) were shown to be affected in hippocampus and prefrontal cortex following chronic or acute stress exposure to^9–15^. In contrast, most studies did not find any changes in GluA2/*Gria2* levels following exposure to stress^10,12,14–16^.

A commonly used model to test for depressive-like behavior in mice is the forced swim test, in which mice are exposed to forced swimming for one or two days^17^. Mice commonly show experience-dependent reduction in mobility on the second day of forced swimming^17,18^. We previously showed that mice lacking GluA1, either globally or selectively in hippocampus, display impaired experience-dependent reduction in mobility on a two-day FST, suggesting an important contribution of hippocampal GluA1-containing AMPA receptors to plastic changes relating to this type of behavior^18^. This impairment was replicated in mice with a mutation of the most C-terminal amino acid (leucine)^18^, which is part of a type I PDZ interaction motif required for direct PDZ-mediated interaction of GluA1-containing AMPA receptors with different postsynaptic proteins, including SAP97^19,20^. However, it is unclear how these proteins specifically contribute to this type of behavior or if the expression of these proteins (or their mRNA) is affected by exposure to forced swim stress.

Thus, in this study, we tested, whether hippocampal gene or protein levels of the two major AMPA receptor subunits GluA1 and GluA2 (encoded by *Gria1* and *Gria2* respectively), as well as the membrane-associated guanylyl kinases (MAGUKs) SAP97 and PSD-95 (encoded by *Dlg1* and *Dlg4* respectively) are affected by exposure to forced swim stress. As the experience-dependent expression of behavioral despair is dependent on GluA1-containing AMPA receptors and their interaction with PSD proteins, we hypothesized that this may be caused at least in part by changes in the expression levels of these proteins following forced swim stress, at least after repeated exposure.

## Materials and Methods

### Mice

A total of 30 eight-week-old female C57BL/6 J mice (Taconic Farms) were used in accordance with the National Institutes of Health Guide for Care and Use of Laboratory Animals. The experimental protocols were approved by the University of Southern California Institutional Animal Care and Use Committee (IACUC #11467 and #11468). Mice were housed individually and kept on a 12-hour light/dark cycle (lights on at 7:00 am) and had *ad libitum* access to food and water. All behavioral experiments were performed during the light phase and after >1 h acclimation to the testing room.

### Forced swim test

The forced swim test was performed as previously described^18^. In brief, mice were exposed to one (FST1, N = 10) or two (FST2, N = 11; one of the mice was removed from the analysis after the first swim session due to a video recording failure; reducing the N to 10) sessions of forced swimming (day 1: 15 min, day 2: 10 min; intersession interval: 24 h) in a white plastic chamber (Ø 30 cm) filled with water (25 ± 1 °C). After the test, mice were dried using a paper towel, returned to their home cages, and placed on a warm plate to dry for 10–15 min to avoid hypothermia^21^.

As previously performed^18^ mouse movement was registered from top view by a USB 2.0 camera at 30 Hz and behavior was analyzed off-line by three human observers independently using Anymaze v6.06 (Stoelting Co). For comparison the automated tracking in Anymaze with the sensitivity for immobility set to 50% and the minimum immobility period set to 1 s was used. For both, supervised and automated tracking, latency to immobility and total immobility during the first five minutes of testing were statistically analyzed.

### RNA isolation and cDNA synthesis

Mice were killed 2 h after FST1 or FST2 by brief exposure to isoflurane followed by decapitation. Untreated and unhandled wild type mice served as controls (CTRL, N = 9). The hippocampus from the right hemisphere was isolated in ice-cold RNAse-free PBS, homogenized in 1 ml TRIzol (Invitrogen) and frozen at −80 °C. RNA was isolated according to the manufacturer’s instruction and treated with DNAse I (Sigma-Aldrich) and 1 µl of each sample was run on an RNA gel (Lonza) to control for RNA integrity. One of the FST1 samples and two of the FST2 samples were removed from further analysis due to RNA degradation. Two additional FST2 samples were removed from further analysis as RNA concentration was insufficient for reverse transcription. All other RNA samples were reverse transcribed with SuperScript III reverse transcriptase (Invitrogen) using 25 ng random hexamers and 5 µM oligo(dT)_20_ on 800 ng RNA/sample.

### Quantitative PCR

Target-specific quantitative PCR (qPCR) was performed in 20 µl reactions containing 10 µl SsoFast™ EvaGreen® Supermix With Low Rox (Bio-Rad), 1 µl target-specific primer and 9 µL prediluted (1:1,000) cDNA using the CFX96 real time PCR cycler (Bio-Rad). All reactions were run in duplicates. PCR conditions: 30 s at 95 °C, followed by 40 cycles of 5 s at 95 °C, 30 s at 58–65 °C (primer dependent, see Table S1 for details) followed by a plate read. At the end of amplification, a melting curve was generated. PCR products of the standard curve were validated on a 2.2% DNA-Agarose gel (Lonza) (Fig. S1). Cycle threshold (Ct) values and amplification curves were obtained using the CFX manager software (version 3.0; Bio-Rad). Data was analyzed using GenEx6 v3.1.3 (MultiD Analyses AB).

One of the FST1 samples was removed from all qPCR analyses as Ct values for all assays were too low compared to the other samples (>4 Cts from the average).

### Protein isolation and preparation of synaptoneurosomes

Hippocampi from the left hemispheres of the same mice used for qPCR were isolated and placed on dry ice immediately and stored at −80 °C. Hippocampi were thawed on ice and homogenized in 1.5 ml 10 mM HEPES (pH 7.4), 1 mM EDTA, 2 mM EGTA, 0.5 mM DTT containing proteinase inhibitors (Complete, EDTA-free, Roche). Of the homogenates 100 µl were kept and SDS was added to a final concentration of 1% and samples were incubated at 72 °C for 5 min. From the rest of the homogenate, synaptoneurosomes were isolated as described in Vilasana *et al*.^22^ using sequential filtration with 100 µm nylon mesh (Millipore) and 5 µm Versapor (PALL) syringe filters followed by centrifugation at 3,600 × *g*. Supernatants were removed and the remaining pellet was dissolved in 100 µl 1% SDS 10 mM HEPES (pH 7.4), 1 mM EDTA, 2 mM EGTA, 0.5 mM DTT containing proteinase inhibitors and incubated at 72 °C for 5 min. The protein concentration of all samples was determined by photometry using the BIORAD protein assay and samples were stored at −80 °C.

### Western blots

For western blotting 10 µg of protein/sample was mixed with Tris-Glycine SDS Sample Buffer (Novex, Invitrogen) containing 2-mercaptoethanol (final concentration: 2.5%). Samples were incubated at 72 °C for 10–15 min, resolved on precast Tris-Glycine protein gels (Novex, Invitrogen) and transferred on PVDF membranes using an iBlot (Invitrogen).

After blotting, membranes were dried for 1 h and then reactivated in methanol and blocked for 1 h in Odyssey blocking buffer (LI-COR), followed by incubation for 1 h in 50% Odyssey blocking buffer (diluted with 1xPBS) containing 0.1% Tween-20 and primary antibodies: *For multiplexing against GluA1 and PSD-95:* Rabbit anti-GluA1 1:1,000 (Millipore AB1504, Lot#1740049), mouse anti-PSD-95 1:10,000 (NeuroMab K28/43, Lot#75–028), mouse anti-GAPDH 1:10,000 (Sigma-Aldrich G8795, Lot#010M4814), mouse anti-β-actin 1:1,000 (Santa-Cruz sc-47778, Lot#C1510). *For staining against SAP97:* mouse anti-SAP97 1:500 (NeuroMab K64/15, Lot#75-030), mouse anti-GAPDH 1:10,000 (Sigma-Aldrich G8795, Lot#010M4814), mouse anti-β-actin 1:1,000 (Santa-Cruz sc-47778, Lot#C1510).

Following primary antibody incubation, membranes were washed six times with 1xPBS containing 0.1% Tween-20 and incubated for 30 min in 50% Odyssey blocking buffer containing 0.1% Tween-20 and secondary antibodies: *For multiplexing against GluA1 and PSD-95:* anti-rabbit IRDye 800CW 1:15,000 (LI-COR 926-32211, Lot#C00503-01), anti-mouse IRDye 680 1:15,000 (LI-COR 926-32220, Lot#C00215-01). *For staining against SAP97:* anti-mouse IRDye 680 1:15,000 (LI-COR 926-32220, Lot#C00215-01).

Membranes were washed six times with 1xPBS containing 0.1% Tween-20, rinsed 2–3 times in 1xPBS and stored in 1xPBS at 4 °C.

### Imaging and analysis

One day after staining, membranes were imaged on the LI-COR Odyssey imaging platform. Images were quantified using ImageStudioLite version 5.2.5 (LI-COR). Specifically, after noise removal, target-specific bands were selected, and signal densities (corrected with the median background 3 px above and below the marked band) were extracted. Values for each target were normalized to the median of that target for each membrane.

For each sample, at least three replicates were run and evaluated. Enrichment of synaptoneurosomes was validated by calculating the ratio of synaptoneurosomal PSD-95 to the PSD-95 levels in homogenates (Fig. S2). For quantification, normalized values for SAP97, PSD-95 and GluA1 were divided by the mean of the respective normalized GAPDH and β-actin values. Values for each sample were averaged after removing extreme outliers (defined as any value above 3 times the interquartile range) and were used for quantification. Samples for which less than three replicates could be quantified (e.g. due to noisy signals), were removed from analysis. Thus, 7 FST1 and 3 FST2 homogenate samples as well as 3 FST2 synaptoneurosome samples were removed from the SAP97 analysis. No samples were removed from the PSD-95 or GluA1 analyses.

### Statistical methods

Analysis of variance (ANOVA) was used for group comparisons and significant effects were analyzed by *post-hoc* Holm-Bonferroni corrected t-tests. In the case of unequal variances, the ANOVA was performed with Welch correction and Dunn’s post-hoc test with Holm-Bonferroni correction. For two group comparison, we made use of independent or paired sample t-tests. In the case that data failed tests for equality of variances and/or normality, the Mann-Whitney-U (independent samples) or Wilcoxon signed rank test (paired samples) was used. Regardless of the type of the test chosen, uncorrected alpha (desired significance level) was set to 0.05 (two-tailed). Statistical analyses were performed using JASP v0.10.

## Results

**Figure 1:**
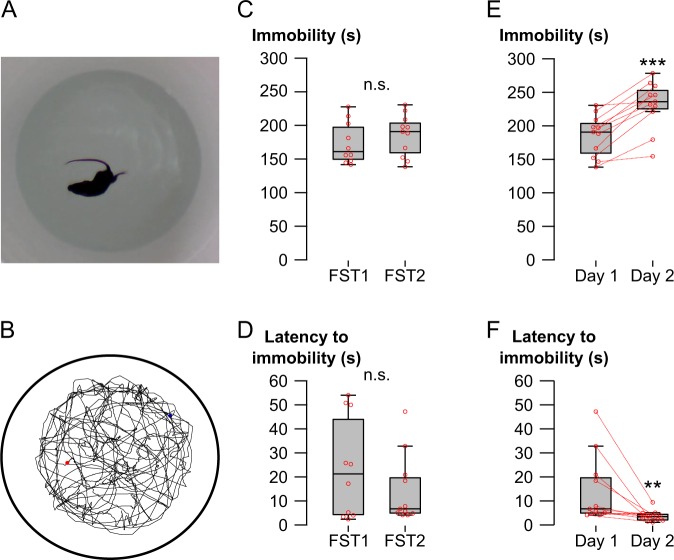
Quantification of behavior in the forced swim test. (**A**) Exemplary video still of a mouse exposed to the swim test apparatus. (**B**) Exemplary track plot of a mouse after 15 min of forced swimming. (**C**–**F**) Behavior was analyzed by human observers (average of three independent observers blind to the treatment). Overall immobility during the first 5 min of swimming (**C**) and latency to immobility (**D**) on day 1 of forced swimming were not significantly different between mice exposed to one day of forced swimming (FST1, N = 10) compared to mice exposed to two days of forced swimming (FST2, N = 11). In FST2 mice, overall immobility during the first 5 min of swimming (**E**) was significantly increased and latency to immobility (**F**) was significantly reduced on day 2 in comparison to day 1. Red circles indicate individual data points. Data points from the same individuals in E and F are connected by a dotted red line. Asterisks indicate statistical significance compared to day 1: **P < 0.01, ***P < 0.001 and n.s. indicates lack of significance.

To test if forced swim exposure results in changes to *Dlg1*, *Dlg4*, *Gria1*, or *Gria2* transcript levels, we subjected C57BL/6 J wild type mice to one (FST1) or two (FST2) sessions of forced swimming (Fig. 1). Swimming behavior in the first 5 min of testing was manually quantified by three human observers, which showed high levels of correlation (Fig. S3), and their results were averaged.

Comparing the behavior of FST1 to FST2 mice during the first forced swim session we found no significant changes in overall immobility (t(19) = −0.87, P = 0.395) (Fig. 1C) or latency to immobility (U = 62, P = 0.647) (Fig. 1D). FST2 mice displayed an experience dependent increase in immobility (t(10) = −6.431, P < 0.001) (Fig. 1E) and reduction in latency to immobility (Z = 63, P = 0.005) (Fig. 1F) as previously shown^18^. The automated analysis using the tracking algorithm in Anymaze was highly correlated with the data from human observers (Fig. S3) and showed comparable results (Fig. S4), further validating the behavioral data from human observers.

**Figure 2:**
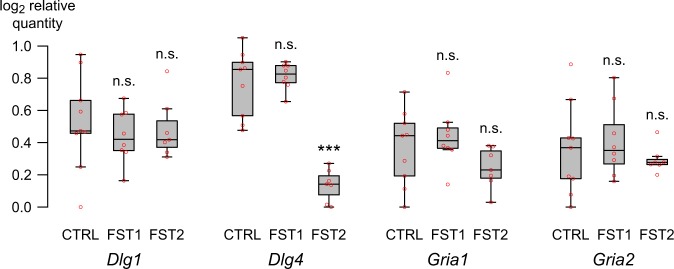
Exposure to the forced swim test for two consecutive days drastically reduces *Dlg4* transcription. Hippocampal expression of *Dlg1*, *Dlg4*, *Gria1*, and *Gria2* mRNA (coding for SAP97, PSD-95, GluA1, and GluA2 respectively) relative to two reference genes (*Pgk1* and *B2m*) after one (FST1; N = 8) or two (FST2; N = 7) swim test exposures relative to untreated wild type mice (CTRL; N = 9) (see Table S1 and Fig. S1 for details on primer pairs). Red circles indicate individual data points. Asterisks indicate statistical significance (Holm-Bonferroni corrected) compared to CTRL mice: ***P < 0.001 and n.s. indicates lack of significance compared to CTRL.

To investigate, whether forced swim stress has an acute effect on gene expression of the genes encoding for SAP97, PSD-95, GluA1 or GluA2 (encoded by *Dlg1*, *Dlg4, Gria1*, and *Gria2* respectively), we performed qPCR experiments using primer pairs targeting all known transcript variants of the respective genes (see Table S1 for primer information) on hippocampal RNA isolated two hours after forced swimming in FST1 and FST2 mice (Fig. 2). We did not find any statistical differences in expression levels for *Dlg1* (F_2,21_ = 0.296, P = 0.747), *Gria1* (F_2,21_ = 1.945, P = 0.168), or *Gria2* (F_2,21_ = 0.504, P = 0.611). In contrast, *Dlg4* expression was significantly affected (F_2,13.191_ = 100.13, P < 0.001), with post-hoc tests showing a significant reduction in *Dlg4* expression in FST2 mice compared to CTRL or FST1 mice (P < 0.001), but no difference between CTRL and FST1 mice (P = 0.445).

**Figure 3:**
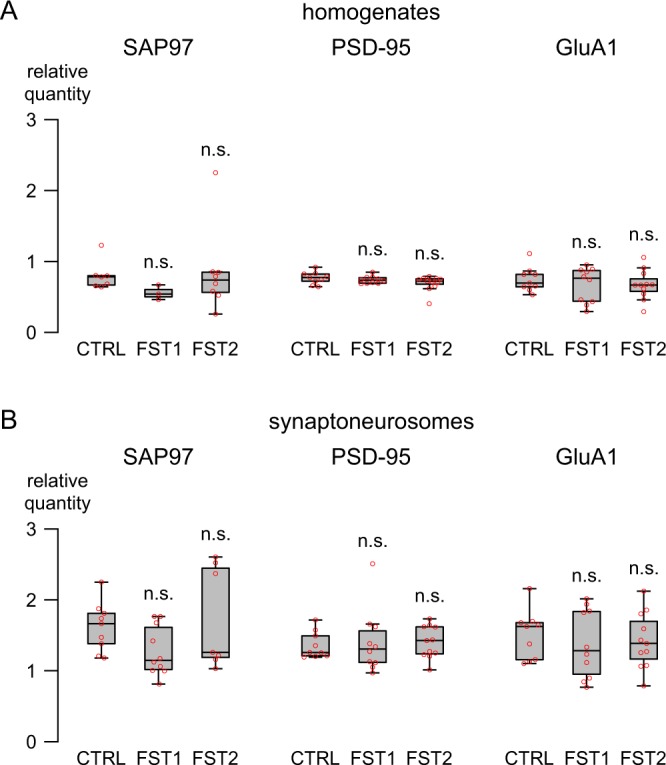
Forced swim stress does not significantly alter protein levels in hippocampus. (**A**) Hippocampal SAP97, PSD-95, and GluA1 levels relative to β-actin and Gapdh after one (FST1; N = 3 for SAP97 homogenates, N = 10 for all others) or two (FST2; N = 7 for SAP97 synaptoneurosomes, N = 8 for SAP97 homogenates, N = 11 for all others) swim test exposures relative to untreated wild type mice (CTRL; N = 7 for SAP97 homogenates, N = 9 for all others). (**B**) Protein levels (relative to β-actin and Gapdh) in synaptoneurosomal fractions prepared from hippocampal homogenates (CTRL, N = 9; FST1, N = 10; FST2; N = 7–11). Note that differences in sample size are a result of samples being removed when the number of available replicates was below 3. Red circles indicate individual data points. Lack of significance compared to CTRL is indicated by n.s.

To identify whether protein levels would be acutely affected by swim stress we analyzed levels of SAP97, PSD-95 and GluA1 (Fig. 3). In hippocampal homogenates, we did not observe any significant changes in the level of these proteins (SAP97: F_2,15_ = 0.507, P = 0.612; PSD-95: F_2,27_ = 1.948, P = 0.162; GluA1: F_2,27_ = 0.421, P = 0.661; Fig. 3A). Levels of PSD-95 and GluA1 were also not affected in synaptoneurosomal fractions (PSD-95: F_2,27_ = 0.058, P = 0.944; GluA1: F_2,27_ = 0.228, P = 0.798; Fig. 3B). However, for SAP97 we could observe a trend for a significant effect in synaptoneurosomes (ANOVA: F_2,12.678_ = 2.661, P = 0.108; Fig. 3B) with post-hoc tests showing a trend towards lower expression in FST1 mice (P = 0.078), but no change in FST2 mice (P = 0.422), suggesting only a transient reduction in SAP97 levels, which is agreement with the observed stable expression of the SAP97 gene (i.e. *Dlg1*) following forced swim stress.

## Discussion

In this study we could show that two-day exposure to forced swimming results in a strong reduction of hippocampal *Dlg4* levels. For all other investigated target mRNAs (i.e. *Dlg1*, *Gria1*, and *Gria2*) or proteins (i.e. PSD-95, SAP97, and GluA1), we did not observe any changes in expression levels.

Stress exposure has been strongly linked to neuroplastic changes in the brain^2,5–7^, which involves changes in synaptic plasticity and memory formation^7^ and exposure to the forced swim test has been linked to these processes^18,23^. Consequently, molecules and pathways involved in neural plasticity are altered following stress exposure. For example, exposure to chronic stress has been shown to reduce hippocampal PSD-95 protein levels (e.g.^24–26^). Interestingly, a study in rats showed only mildly reduced levels of PSD-95 protein following forced swimming for two sessions^27^. This is well in line with our finding that repeated exposure to forced swimming resulted in reduced expression of the PSD-95 gene (i.e. *Dlg4*), while protein levels were not affected at that time point. It is conceivable that a reduction in PSD-95 protein levels may only be detectable later following stress exposure, which, however, was not assessed in the present study.

Much less is known about the role of SAP97 in response to stress. However, as SAP97 is importantly involved in synaptic plasticity and learning and memory, in part through the interaction with GluA1^20,28^, SAP97 is most likely involved in the stress response. Our previous findings directly link the interaction between GluA1 and SAP97 to forced swim behavior^18^. However, our findings in the present study do not suggest a significant change in synaptic levels of SAP97 following forced swim stress, suggesting that this protein may be less directly involved in mediating the stress response following forced swimming.

Concerning GluA1, our results are in contrast with previous observations showing changes in AMPA receptor subunit expression (particularly of *Gria1*/GluA1) as a consequence of acute or chronic stress (reviewed in^8^). The GluA1 subunit of AMPA receptors is critically involved in AMPA receptor-dependent synaptic plasticity, leading to fast alterations of AMPA receptor-mediated signaling by different mechanisms, including changes in gene transcription, protein translation, protein transport and posttranslational modification (see e.g.^6,29,30^ for review). Thus, GluA1 may be altered by one or more of these mechanisms following forced swim stress.

The lack of changes in synaptoneurosomes may suggest that transport of GluA1 to or from the synapse may not be affected in our experiments. However, as synaptoneurosomal preparations still contain relatively large portions of the postsynaptic membrane^22,31,32^, changes in local receptor trafficking may not be observable, but may require isolation of postsynaptic densities. In fact, a recent study performed in male mice could show that GluA1 levels were elevated following acute forced swim exposure in postsynaptic densities prepared from hippocampus, but, analogous to our findings, failed to observe any changes to GluA1 levels in total homogenates^33^.

We cannot exclude the possibility that forced swim stress may have resulted in posttranslational modification of AMPA receptors. For example, phosphorylation of GluA1 at serine831 and serine845 has been shown to be important for synaptic plasticity^34,35^ and is changed following stress exposure^36^. Moreover, GluA1 is phosphorylated at these sites following treatment with classical antidepressants^37,38^. In fact, the study by Ai *et al*.^33^ showed that forced swim exposure increases phosphorylation of GluA1 at Ser845. Other posttranslational mechanisms that may contribute to the involvement of AMPA receptors in forced swim behavior may involve acetylation and ubiquitination, both of which have been shown to be altered in GluA1 and GluA2 subunits following stress^39,40^.

There are some limitations to this study: First, we only investigated a limited number of genes/proteins. While the study was designed as such, since we expected to see changes in these genes based on our previous findings in GluA1 knockout mice^18^ as well as from findings in other studies, that suggested an important role of AMPA receptors in stress and depression^8^, it is more than likely that other targets will be effected. For example, expression of BDNF, mGluR5 and GluN1, among others, has been shown to be altered following forced swim stress^27,41,42^. Moreover, while we did not find changes in *Gria2* levels, which is consistent with most studies investigating mRNA levels of this gene following stress exposure^12,14,16^, we did not measure protein levels of GluA2. While one study found a reduction in surface GluA2 in prefrontal cortex, following chronic restraint stress^43^, another study investigating GluA2 levels in CA1 did not find any changes following chronic mild stress^10^. Thus, in the present study, it is possible that we have missed potential changes in GluA2 protein levels following swim test exposure.

Second, we only investigated two time points, i.e. 2 hours after one (FST1) or two (FST2) sessions of forced swimming. Long-term changes caused by the forced swim stress could thus not be fully assessed. Further investigations should focus on long-term effects of only one forced swim exposure, without the added effect of a second forced swim session, as well as the long-term effects following two sessions of forced swimming. Moreover, we assessed both mRNA and protein at the same time points, although their expression and degradation dynamics may not correlate in response to perturbations^44^ such as swim stress.

Third, in this study we only investigated effects on hippocampal gene/protein expression since our previous findings suggested that AMPA receptors in hippocampus are essential for the experience dependent induction of behavioral despair^18^. However, in other studies acute or chronic stress exposure resulted in changes in AMPA receptor levels also in other brain regions, such as the entorhinal cortex, nucleus accumbens, ventral tegmental area and prefrontal cortex^11,13,43^. Moreover, as described above, the study by Wang *et al*.^27^ only found mild effects of forced swimming on PSD-95 expression in hippocampus, but a significant increase in PSD-95 levels in prefrontal cortex. Thus, it is possible that the observed genes/proteins may be altered in regions outside of the hippocampus.

Finally, we only investigated female mice. This was done, as in our previous study^18^ we could show that experience dependent reduction in immobility was reliably observed only in females. Nevertheless, sex-dependent differences in forced swim behavior have been reported (for review see^45,46^). Moreover, it has been shown that male and female rodents respond differentially to acute and chronic stress in terms of their endocrine as well as neurobiological response (for review see e.g.^46–48^). With regards to AMPA receptors it has been shown that following swim stress exposure, [^3^H]AMPA binding was reduced in the forebrain of male but not female mice^49^. Stress from maternal separation was shown to reduce prefrontal GluA2 levels in male but not female adolescent rats^50^. Moreover, fluctuations in [^3^H]AMPA binding and AMPA receptor stoichiometry have been shown during different stages of the estrous cycle in rats^51,52^. Of note, the estrous stage of the mice tested in this study has not been determined. Thus, we cannot exclude the possibility that swim stress exposure in males would result in differential changes than those observed in female mice herein.

In summary, our study shows that in female mice the expression of *Gria1*/GluA1 is not acutely affected by exposure to the forced swim test, suggesting that GluA1 is modified by other non-genomic mechanisms thereby contributing to forced swim behavior. Moreover, we found a transient effect for reduced SAP97 levels, and a strong reduction in PSD-95 gene expression after repeated forced swim exposure suggesting more permanent changes in PSD-95, which, however, will have to be validated in future studies.

## Supplementary information

Supplementary Figures and Tables
