# Supplementary material for: Quantitative analysis of Gria1, Gria2, Dlg1 and Dlg4 expression levels in hippocampus following forced swim stress in mice
Source: Sci Rep. 2019 Oct 1;9:14060. doi: 10.1038/s41598-019-50689-w (PMC6773768; doi:10.1038/s41598-019-50689-w)
Supplement: Supplementary file 1 — Supplementary Figures and Tables [file 41598_2019_50689_MOESM1_ESM.docx]

**Supplementary Material**

Quantitative analysis of Gria1, Gria2, Dlg1 and Dlg4 expression levels in hippocampus following forced swim stress in mice

Florian Freudenberg^a,b,*^

^a^ Department of Psychiatry, Psychosomatic Medicine and Psychotherapy, University Hospital, Goethe University, Frankfurt, Germany

^b^ Laboratory of Neural Circuits and Plasticity, University of Southern California, 3641 Watt Way, Los Angeles, CA-90089, USA

**Table S1: Primer pairs used for qRT-PCR**

| **Name (Target)^a^** | **Sequences^b^** | **Target region^c^** | **PCR^d^** | **Standard curve^e^** |
| --- | --- | --- | --- | --- |
| *Rpl13a* (Mus musculus ribosomal protein L13A) | F: ATGACAAGAAAAAGCGGATG  R: CTTTTCTGCCTGTTTCCGTA | 366-580 of NM_009438.5 | 500 nM  60 ºC | y = -3.62157x+17.69059  r^2^ = 0.99286  E = 0.88853 |
| *Pgk1* (Mus musculus phosphoglycerate kinase 1) | F: GCAGATTGTTTGGAATGGTC  R: TGCTCACATGGCTGACTTTA | 1147-1331 of NM_008828.3 | 250 nM  62 ºC | y = -3.6602x+19.16893  r^2^ = 0.99073  E = 0.8759 |
| *B2m* (Mouse beta-2 microglobulin) | F: GGCCTGTATGCTATCCAGAA  R: GAAAGACCAGTCCTTGCTGA | 100-297 of NM_009735.3 | 500 nM  58 ºC | y = -3.61698x+20.4924  r^2^ = 0.9946  E = 0.89005 |
| *Dlg1* (Mus musculus discs large MAGUK scaffold protein 1 [SAP97]) | F: CAGGACTAACCATGGAGGTG  R: CCCCTCCCTAAGCATACATT | 3805-4017 of NM_007862.3, 3703-3915 of NM_001252433.1, 3640-3852 of NM_001252434.1, 3398-3610 of NM_001252435.1, 3541-3753 of NM_001252436.1,  3471-3683 of NM_001357281.1 and 3661-3873 of NM_001357282.1 | 250 nM  58 ºC | y = -3.3678x+22.50361  r^2^ = 0.99653  E = 0.98121 |
| *Dlg4* (Mus musculus discs, large homolog 4 [PSD-95]) | F: GGGCCACGAAGCTGGAGCAG  R: CCAGGTGGTGGAGGCAGGGT | 2342-2536 of NM_007864.3 and 2333-2527 of NM_001109752.1 | 250 nM  65 ºC | y = -3.39385x+19.00285  r^2^ = 0.99328  E = 0.97084 |
| *Gria1* (Mus musculus glutamate receptor, ionotropic, AMPA1 (alpha 1) [GluA1]) | F: CTAGGCTGCCTGAACCTTTG  R: GGGAAGATTGAATGGAAGCA | 4641-4849 of NM_001113325.2 and NM_008165.4, 4268-4476 of NM_001252403.1 | 250 nM  58 ºC | y = -3.52347x+18.8435  r^2^ = 0.99417  E = 0.92226 |
| *Gria2* (Mouse glutamate receptor, ionotropic, AMPA2 (alpha 2) [GluA2]) | F: ATCGAGCAGAGGAAGCCTTGCG  R: GTGGCGATGCCGTAGCCTTTGG | 2648-2721 of NM_001083806.2, NM_013540.3, NM_001039195.2, NM_001357924.1, and NM_001357927.1 | 500 nM  65 ºC | y = -3.32623x+20.77754  r^2^ = 0.99748  E = 0.99821 |

^a^ Name of the primer pair/target gene and full name of the target gene (protein names that differ from the gene name are written in square brackets).

^b^ Nucleotide sequences of the forward (F) and reverse (R) primers.

^c^ Region of the target gene amplified by the primer pair.

^d^ Final concentration (in nM) and Annealing/Extension temperature (in ºC) used for amplification.

^e^ Linear function and coefficient of determination (r^2^) of the standard curve and efficiency (E) of the primer calculated from the slope.

**
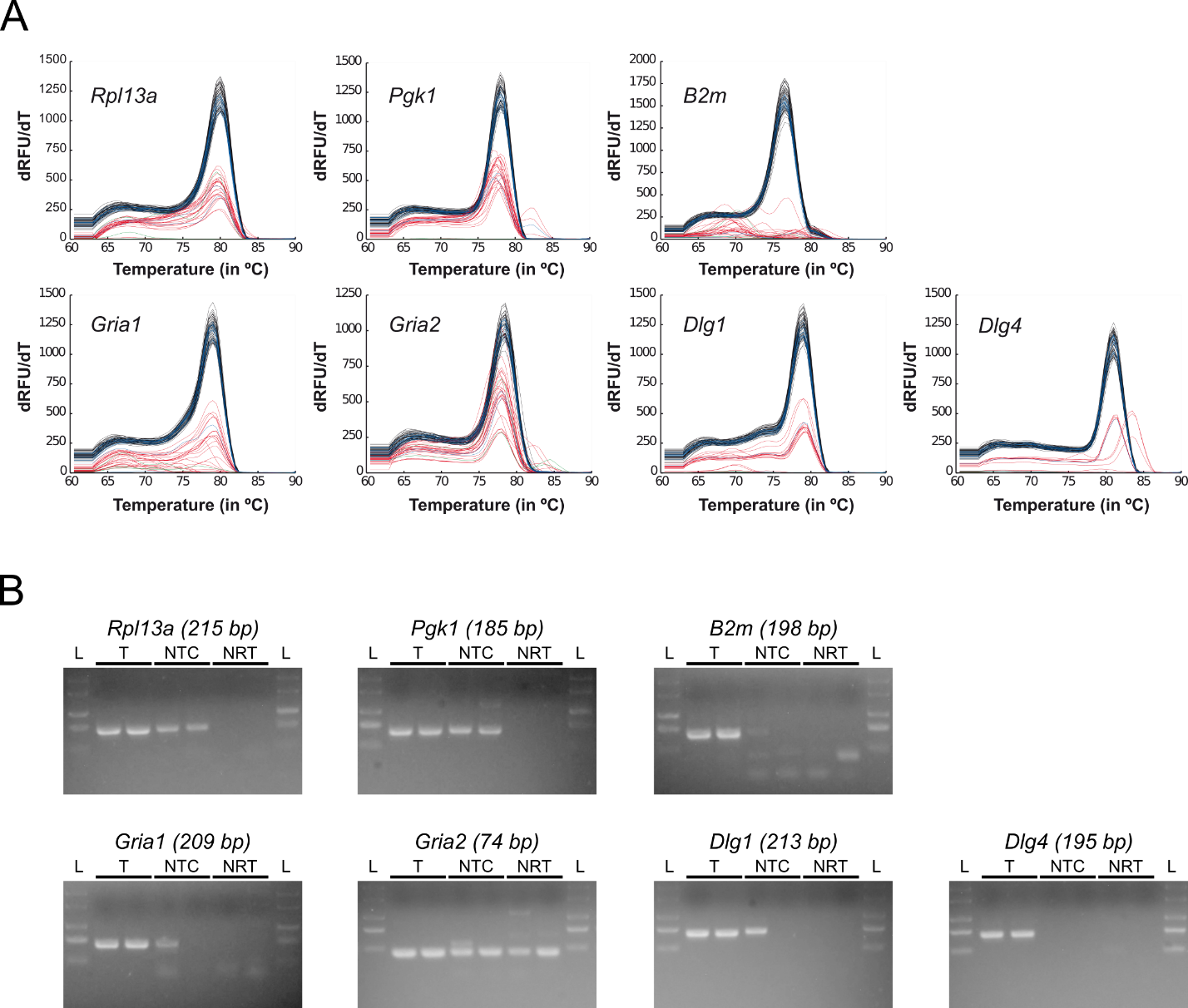
**

**Figure S1 (A)** Melt-peaks (calculated as the quotient of the change in relative fluorescent units (dRFU) and the change in temperature (dT)) for all primer pairs used in this study. Target sample traces are shown in black, non-reverse transcription (NRT) controls are shown in red, non-target controls (NTC) are shown in green, and standards are shown in blue. For all genes there is a single distinct peak for the target and standard samples, which is substantially weaker or absent in NRT controls and absent in NTC. This indicates specific amplification of a single target during PCR. **(B)** Representative qRT-PCR samples visualized on a 2.2% Agarose gel. For all primer pairs, there is a single band at the appropriate size (shown in parantheses) for the target samples (T), and for some of the NRT controls, but not for the NTC (except for *Gria2*). In most cases, when controls show a band it is substantially weaker than the target sample band and of different size (L = Lonza Flashgel quantladder [100/250/400/800/1,500 bp]) .


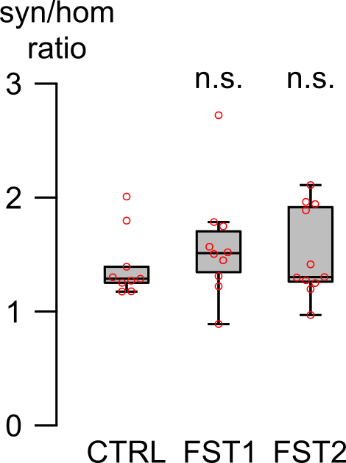


**Figure S2: Validation of synaptoneurosomal enrichment.** Quantification of PSD-95 enrichment in synaptoneurosomal preparations (syn/hom ratio). In control (CTRL), FST1 and FST2 mice PSD-95 was enriched ~1.5x indicating successful synaptoneurosomal enrichment. No statistical differences were detected in the level of enrichment between CTRL, FST1, or FST2 mice (F_2,27_=0.414, P=0.665). Red circles indicate individual data points. Lack of a significant difference compared to CTRL is indicated by the n.s.


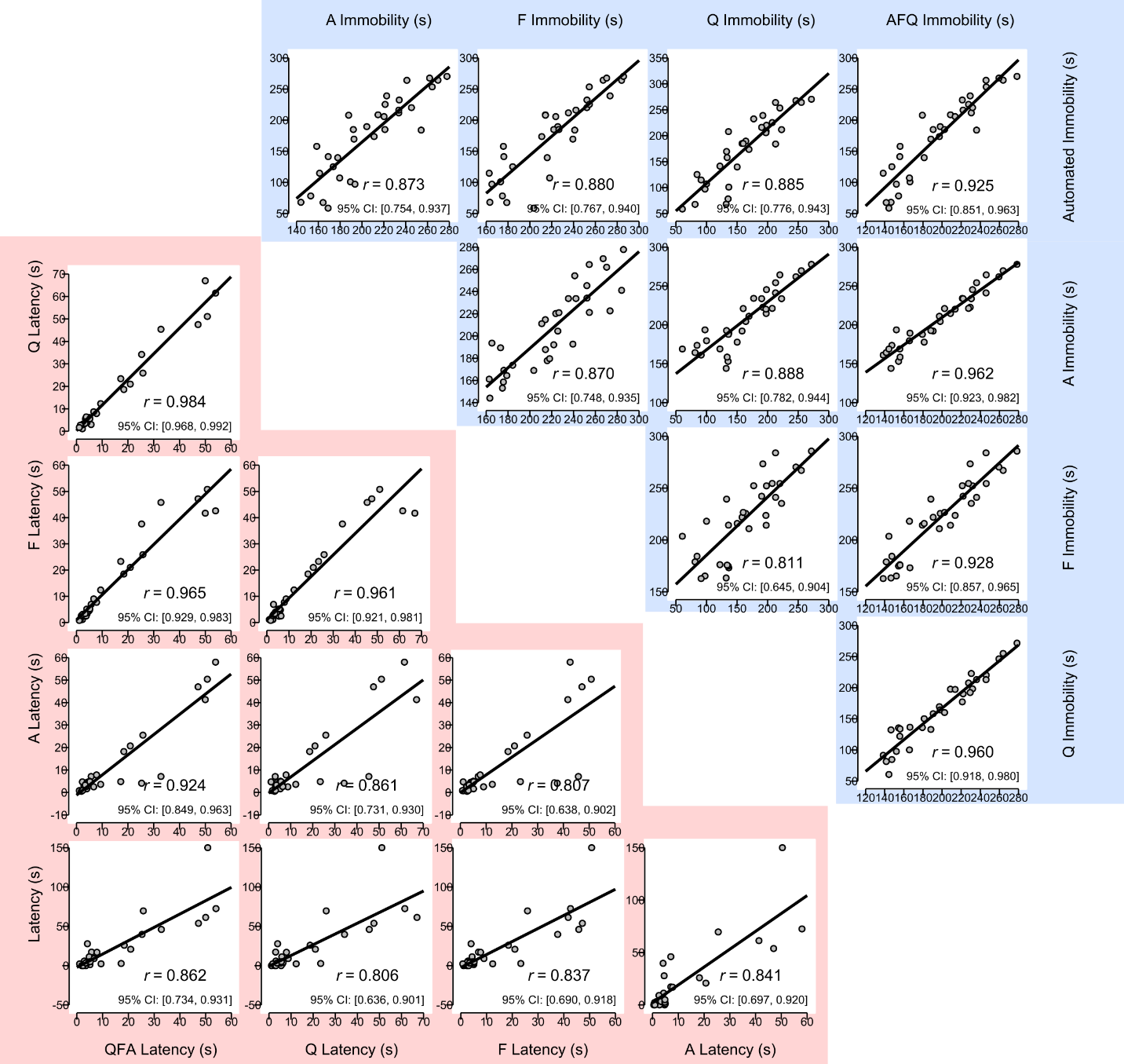


**Figure S3:** Correlation matrices for overall immobility (upper right; blue shading) and latency to immobility (lower left; red shading) comparing the individual measurements of three different human observers (Q, F, and A) and their average (QFA) to the automated analysis performed in Anymaze. All comparisons show highly significant correlations (p<0.001).

**
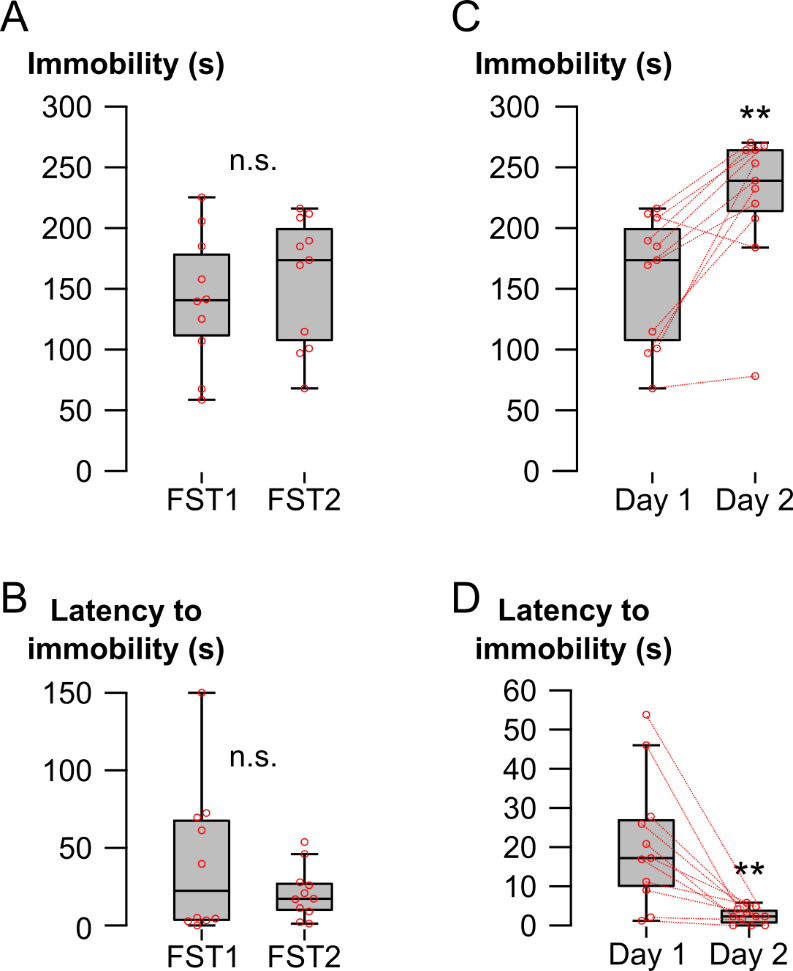
**

**Figure S4: Automated analysis of forced swim test behavior using Anymaze.** As with the analysis from human observers (Figure 1) overall immobility **(A)** and latency to immobility **(B)** on day 1 of forced swimming were not significantly different between mice exposed to one day of forced swimming (FST1) compared to mice exposed to two days of forced swimming (FST2) (Immobility: t(19)=-0.696, P=0.495; Latency to immobility: U=61, P=0.705). In FST2 mice, overall immobility **(C)** was significantly increased and latency to immobility **(D)** was significantly reduced on day 2 in comparison to day 1 (Immobility: t(10)=-4.425, P=0.001; Latency to immobility: t(10)=3.753, P=0.004). Red circles indicate individual data points. Data points from the same individuals in E and F are connected by a dotted red line. Asterisks indicate statistical significance compared to day 1: **P<0.01 and n.s. indicates lack of significance.
